# Weighing in on Adipogenesis

**DOI:** 10.3389/fphys.2022.821278

**Published:** 2022-02-25

**Authors:** Elizabeth R. Nunn, Abhijit B. Shinde, Elma Zaganjor

**Affiliations:** Department of Molecular Physiology and Biophysics, Vanderbilt University School of Medicine, Nashville, TN, United States

**Keywords:** epigenetics, mitochondria, metabolism, adipose, adipogenesis

## Abstract

Obesity is a growing health concern worldwide because of its contribution to metabolic syndrome, type II diabetes, insulin resistance (IR), and numerous cancers. In obesity, white adipose tissue (WAT) expands through two mechanisms: increase in adipocyte cell number by precursor cell differentiation through the process of adipogenesis (hyperplasia) and increase in existing mature adipocyte cell size (hypertrophy). While hypertrophy is associated with the negative effects of obesity on metabolic health, such as inflammation and lipotoxicity, adipogenesis prevents obesity-mediated metabolic decline. Moreover, in metabolically healthy obesity adipogenesis is increased. Thus, it is vital to understand the mechanistic basis for adipose expansion to inform novel therapeutic approaches to mitigate the dysfunction of this tissue and associated diseases. In this mini-review, we summarize recent studies on the regulation of adipogenesis and provide a perspective on targeting adipogenesis as a potential therapeutic avenue for metabolic disorders.

## Introduction

Although adiposity is linked to numerous diseases, adipocytes are critical for maintaining healthy systemic metabolism. Adipocytes perform important functions: coordinating energy balance, releasing nutrients in times of fasting, and storing nutrients in times of surplus. WAT is an endocrine organ that controls appetite, fertility, body temperature, glucose homeostasis, and insulin sensitivity by secreting hormones (adipokines), cytokines, and lipids. Adipose serves physical functions, such as cushioning organs like the heart, kidneys, ovaries, and extremities that experience high levels of stress, like the palms and heels. WAT expands through hyperplasia, an increase in adipocyte cell number through precursor cell differentiation *via* a process termed adipogenesis, and hypertrophy, an increase in existing, mature adipocyte cell size. Hypertrophy is accompanied by inflammation, lipotoxicity, and ectopic lipid accumulation, which may lead to IR. In contrast, adipogenesis prevents obesity-mediated metabolic decline. Genetically engineered mouse models which exhibit increased adipogenesis in the subcutaneous adipose depot are metabolically healthy despite increased adiposity (Kim et al., 2007; Kusminski et al., 2012). Therefore, inducing adipogenesis and limiting hypertrophy could present a therapeutic strategy for combating metabolic diseases induced by overnutrition. What are the molecular mechanisms that promote adipogenesis? Various signaling pathways and transcriptional and epigenetic regulators of adipogenesis have been elucidated. However, how cellular metabolism mechanistically regulates adipogenesis is only now being evaluated. In this mini-review, we summarize the literature on the role of cellular metabolism in adipogenesis and discuss the potential implications for the treatment of obesity-mediated disorders.

## Signaling Pathways in Adipogenesis

Hormones and ligands regulate adipogenesis by modulating signaling pathways. Insulin engages the insulin receptor, activating the signaling cascade consisting of insulin receptor substrate (IRS), phosphoinositide 3-kinase (PI3K), AKT, and the mechanistic target of rapamycin complex 1 (mTORC1) to promote glucose uptake into differentiating preadipocytes. Glucocorticoids bind glucocorticoid receptor (GCR) to stimulate transcription *via* CCAAT/enhancer-binding proteins (C/EBPs) and peroxisome proliferator-activated receptor γ (PPARγ), enhancing insulin signaling required for adipogenesis (Ambele et al., 2020). The bone morphogenic proteins (BMPs), specifically BMP2 and BMP4, promote adipogenesis in part through SMAD4-mediated upregulation of PPARγ (Zamani and Brown, 2011). The hedgehog signaling pathway opposes adipogenesis through interference of BMP signaling (Suh et al., 2006; Garten et al., 2012). Canonical Wnt signaling suppresses adipogenesis through the stabilization of β-catenin that represses the PPARγ-C/EBPα complex (Kanazawa et al., 2005; Rosen and MacDougald, 2006). Other pathways, such as TGFβ, FGF, ERK/MAPK, p38/MAPK, and Notch signaling, can be pro- or anti-adipogenic depending on the experimental system, specific ligands used, and cell type, as well as the developmental or differentiation stage (Bost et al., 2005; Aouadi et al., 2006; Rosen and MacDougald, 2006; Zamani and Brown, 2011).

## Transcriptional Control of Adipogenesis

PPARγ is one of the key transcriptional regulators of adipogenesis. Although early mechanistic studies investigating the function of PPARγ in adipogenesis were performed using *in vitro* model systems (Tontonoz et al., 1994; Rosen et al., 1999), the loss of WAT in PPARγ-deficient mouse models such as chimeric PPARγ-null (Rosen et al., 1999), tetraploid rescued (Barak et al., 1999), and adipocyte-specific PPARγ knockouts (KOs) (Wang et al., 2013a) provide evidence for its role in adipogenesis *in vivo*. The action of PPARγ promotes the transcriptional activity of another adipogenic regulator, co-activator CCAAT/enhancer-binding protein-α (C/EBPα) (Wu et al., 1999). When expressed ectopically, C/EBPα induces adipogenesis in fibroblasts (Freytag et al., 1994). However, C/EBPα-deficient fibroblasts can only undergo partial adipogenesis, displaying several defects, such as decreased lipid accumulation and IR (Wu et al., 1999). The terminal step of embryonic adipogenesis is independent of C/EBPα, possibly due to redundancy with other C/EBP family members, whereas it is essential for various WAT adipogenic conditions during adulthood (Wang et al., 2015). Moreover, PPARγ can induce adipogenesis in the absence of C/EBPα, but C/EBPα fails to do so in PPARγ-deficient fibroblasts (Rosen et al., 2002). These observations suggest that C/EBPα and PPARγ work cooperatively in a single pathway, although PPARγ plays a central effector role in adipogenesis. C/EBPβ and C/EBPδ are expressed earlier during induction of the adipogenic program in 3T3-L1 cells, and they promote the expression of C/EBPα during the final phase of adipogenesis (Cao et al., 1991; Yeh et al., 1995). Additionally, mice lacking both C/EBPβ and C/EBPδ showed a dramatic volume reduction of epidydimal WAT (eWAT) (Tanaka et al., 1997). Apart from these main factors, several other transcriptional regulators of adipogenesis have been identified and are reviewed in-depth elsewhere (Farmer, 2006; Rosen and Spiegelman, 2014; Lee et al., 2019).

## Metabolic Regulation of Adipogenesis

**Figure 1:**
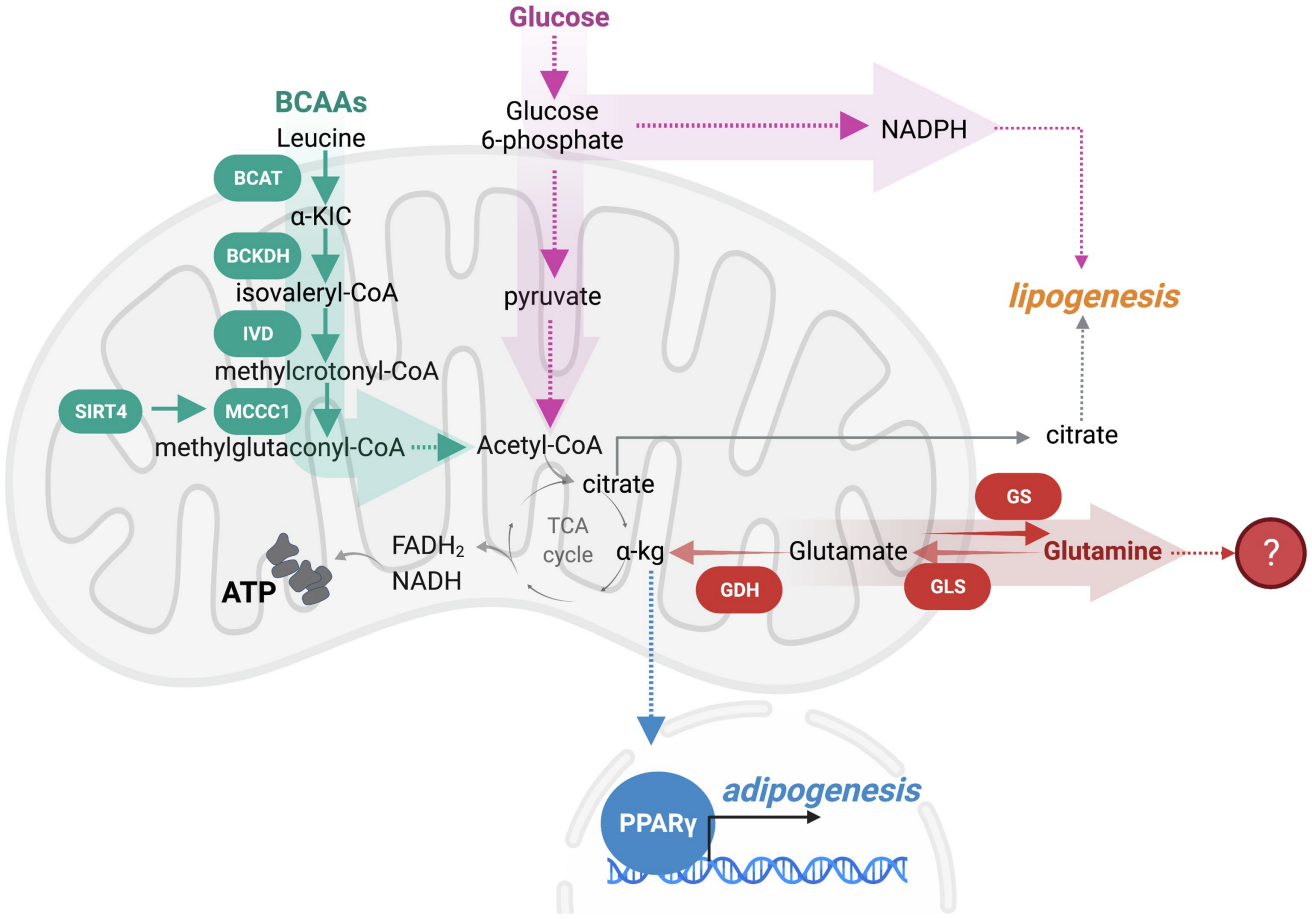
Nutrient oxidation in the mitochondria modulates adipogenesis. Branched-chain amino acids (BCAAs) and glucose catabolism within the mitochondria produce ATP and metabolic intermediates that promote adipogenesis. As such, leucine catabolism is an early regulator of adipogenesis that is initiated by a BCAA transaminase (BCAT) generating α-ketoisocaproic acid (a-KIC). a-KIC is irreversibly oxidized by the BCAA dehydrogenase (BCKDH) complex forming isovaleryl-CoA. Isovaleryl-CoA dehydrogenase (IVD) converts isovaleryl-CoA to methylcrotonyl-CoA. Sirtuin SIRT4 induction of methylcrotonyl-CoA carboxylase (MCCC1) promotes the carboxylation of 3-methylcrotonyl-CoA to 3-methylglutaconyl-CoA, which can be further oxidized to produce acetyl-CoA. BCAA catabolism promotes the PPARγ-mediated transcriptional adipogenic program. While a portion of glucose is oxidized to generate acetyl-CoA, some glucose is diverted away from the mitochondrial oxidation to the pentose phosphate pathway (PPP) to support the production of nicotinamide adenine dinucleotide phosphate (NADPH), a required cofactor for lipogenesis. Glutaminolysis appears to oppose adipogenesis, although the mechanistic understanding of fuel switching that supports adipogenesis is limited.

Given that adipocytes are a reservoir for lipid stores and sense nutrient state to regulate organismal energy balance, metabolites themselves are significant regulators of adipogenesis. Glucose availability dictates adipocyte maturation, such that GLUT4-mediated glucose uptake promotes adipogenesis through a number of distinct mechanisms (Kahn and Flier, 2000). First, glucose contributes to nicotinamide adenine dinucleotide phosphate (NADPH) synthesis through the pentose phosphate pathway. As NADPH is a required cofactor for lipogenesis, glucose directly mediates adipocyte differentiation through this pathway (Jackson et al., 2017). Another functional use of glucose is in acetyl-coenzyme A (acetyl-CoA) production. Preadipocytes derive acetyl-CoA for *de novo* lipogenesis from glucose, with the rate-limiting step in this pathway being catalyzed by pyruvate dehydrogenase (PDH). Upon differentiation, adipocytes decrease glucose contribution to lipogenic acetyl-CoA. To supplement this carbon pool, the catabolism of branched-chain amino acids (BCAAs), leucine, isoleucine, and valine, is another source of lipogenic acetyl-CoA that promotes adipogenesis (Green et al., 2016). Mitochondrial BCAA catabolism involves transamination of BCAAs by the branched-chain amino transferase (BCAT), generating branched-chain α-keto acids (BCKAs). BCKAs are oxidized by the BCAA dehydrogenase (BCKDH) complex. During the remainder of oxidation BCAA carbons are either lost as CO_2_ or contribute carbons to the tricarboxylic acid (TCA) cycle as succinyl-CoA or acetyl-CoA (Neinast et al., 2019a). In adipogenesis, a mitochondrial sirtuin, SIRT4, promotes leucine catabolism by increasing the activity of methylcrotonyl-CoA carboxylase (MCCC1), an enzyme that catalyzes the carboxylation of 3-methylcrotonyl-CoA to 3-methylglutaconyl-CoA (Anderson et al., 2017; Zaganjor et al., 2021). The induction of leucine catabolism occurs early in the process of differentiation and further promotes PPARγ function (Zaganjor et al., 2021). Adipogenesis reduces the contribution of glutamine carbon to fatty acids and instead promotes *de novo* glutamine synthesis (Green et al., 2016). Moreover, knockdown of glutaminase (Gls), which promotes glutaminolysis by deamination of glutamine to glutamate, stimulates adipogenic fate in skeletal stem cells (Yu et al., 2019). Therefore, glutamine oxidation appears inhibitory to adipogenesis, although the mechanism remains elusive. Because almost all the aforementioned nutrient oxidation pathways, other than the pentose phosphate pathway, occur in the mitochondria, these studies demonstrate a vital contribution of mitochondrial function in adipogenesis (Figure 1).

## Mitochondrial Function in Adipocyte Differentiation

Adipogenesis involves a 20- to 30-fold increase in the concentration of mitochondrial proteins as determined by both proteomics and electron microscopy (Wilson-Fritch et al., 2003). Mechanistically, the transcriptional program consisting of cyclic adenosine monophosphate (cAMP) responsive element binding protein (CREB), PPARγ, C/EBPα, estrogen-related receptor α (ERRα), and PPARγ co-activator 1 (PGC-1) are engaged to support mitochondrial biogenesis. Mitochondrial respiratory capacity increases with differentiation, suggesting that mitochondria are biochemically altered (Wilson-Fritch et al., 2003). Successful adipogenesis relies on mitochondrial biogenesis.

Which mitochondrial functions support differentiation? Beyond nutrient oxidation within the mitochondria to generate acetyl-CoA, the mitochondrial electron transport chain (ETC) is a source of reactive oxygen species (ROS) that can also modulate differentiation. ROS can regulate mitochondrial dynamics, increasing mitochondrial fission in bone marrow mesenchymal stem cells (BMSCs) to promote adipogenesis (Guo et al., 2020). While mitochondrial-targeted antioxidants block adipogenesis (Tormos et al., 2011), constitutively high levels of ROS have an inhibitory effect on adipogenesis through the induction of a transcriptional repressor C/EBP homologous protein 10 (CHOP-10) (Carriere et al., 2004; Furukawa et al., 2004). The toxic elevation of ROS also causes structural cellular damage to mitochondrial proteins, lipids, and DNA, including damage to the ETC, which results in depletion of ATP and nicotinamide adenine dinucleotide (NAD) (Rolo et al., 2012). In human clinical trials, antioxidants were not successful in preventing risks of type II diabetes (T2D), possibly because of the beneficial effects of ROS on adipogenesis (Castro et al., 2016). Gaining insight into the threshold of ROS that has beneficial, pro-adipogenic effects may be critical in treating metabolic dysfunction.

## Epigenetic Control of Adipogenesis

**Figure 2:**
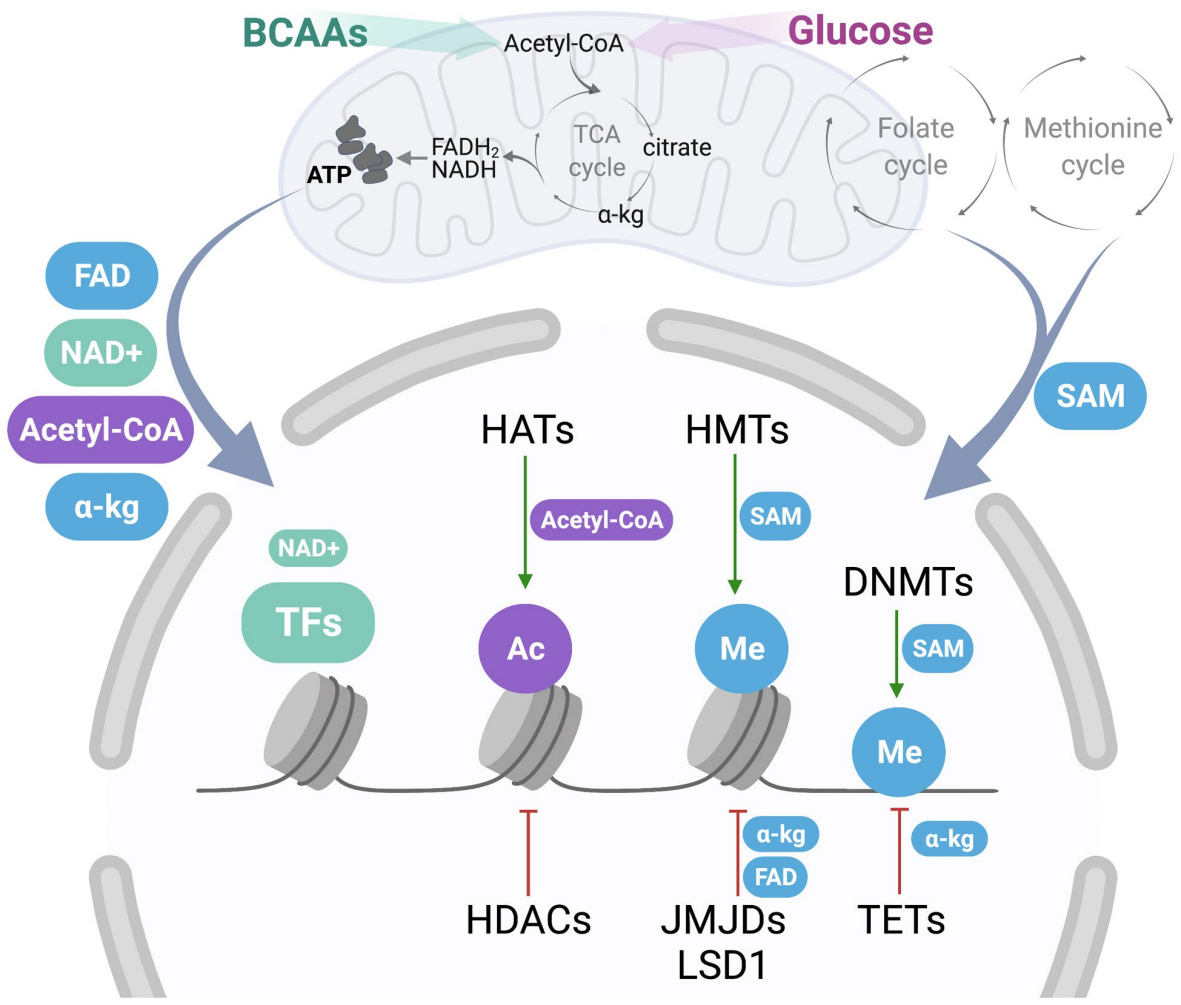
Communication between mitochondria-derived metabolites and nuclear epigenetic regulators. One-carbon metabolism, consisting of folate and methionine cycle, generates S-adenosylmethionine (SAM), a metabolite contributing to epigenetic regulation. Oxidation of fuels, such as BCAAs and glucose, coupled to the activity of the tricarboxylic acid (TCA) cycle and the electron transport chain (ETC) produce important metabolic byproducts, including flavin adenine dinucleotide (FAD), nicotinamide adenine dinucleotide (NAD+), acetyl-coenzyme A (acetyl-CoA), and α-ketoglutarate (a-kg). Once exported out of the mitochondria, these metabolites may contribute to cytosolic signaling, or act as cofactors and donor groups to epigenetic regulators in the nucleus, including histone acetyltransferases (HATs) and deacetylases (HDACs), histone and DNA methyltransferases (HMTs and DNMTs), and demethylases that include ten-eleven translocation DNA demethylases (TETs), and histone demethylases, such as JmjC domain-containing histone demethylases (JMJDs) and histone lysine demethylase 1 (LSD1), as well as other nuclear transcription factors (TFs). HATs add acetyl groups (Ac, in purple) to histones using acetyl-CoA as a substrate, while HDACs remove these groups. HMTs utilize SAM as a donor of a methyl group (Me, in blue) to histones, while methyl groups are removed by JMJDs, which utilize a-kg, or LSD1, which couples with FAD synthesis. Similarly, DMNTs utilize SAM for DNA methylation, while a-kg is a cofactor used by TETs for DNA demethylation. Additionally, NAD+ acts as an important cofactor for nuclear TFs and other regulatory enzymes, including PARP-1 and sirtuins.

Epigenetic modifications including acetylation, methylation, phosphorylation, and ribosylation are regulated by several different classes of enzymes that connect the energetic status of the cell to changes in the epigenetic landscape. Epigenetic regulators may have positive or negative effects on adipogenesis and include histone acetyltransferases (HATs) and deacetylases (HDACs), histone, and DNA methyltransferases (HMTs and DNMTs), and demethylases that include ten-eleven translocation DNA demethylases (TETs), and histone demethylases, such as JmjC domain-containing histone demethylases (JMJDs) and histone lysine demethylase 1 (LSD1) (Musri et al., 2010; Chatterjee et al., 2011; Lee and Ge, 2014) (Figure 2). The epigenetic landscape is complex and multi-faceted, as mono-, di-, and tri-methylation can occur at both histone and DNA methylation marks, and acetylation, phosphorylation, and ribosylation are just a few of the dynamic modifications that can be added and removed by enzymatic regulators (Lee et al., 2019). Epigenetic patterns are altered as preadipocytes commit to this lineage and differentiate into mature adipocytes, and these changes can silence or promote the expression of particular genes as necessary. For example, histone H3K9 di-methylation *via* G9a in the promoter region of *Ppar*γ represses its expression and subsequently blocks differentiation (Wang et al., 2013b). LSD1 decreases H3K9 di-methylation while maintaining H3K4 di-methylation at the *Cebpa* promoter to promote adipogenesis (Musri et al., 2010). LSD1 can also demethylate H3K9 to support adipogenesis through PPARγ activity (Jang et al., 2017). Furthermore, a specific bivalent chromatin signature H3K4/H3K9me3 keeps the expression of *Ppar*γ and *Cebpa* at low levels, allowing preadipocytes to remain primed for differentiation (Matsumura et al., 2015). This complex array of histone and DNA modifications that are added and removed by epigenetic regulators rely on metabolites generated within the mitochondria.

## Metabolic Regulation of Epigenetics in Adipogenesis

Metabolic intermediates and cofactors, including acetyl-CoA, α-ketoglutarate, and NAD+, are derived in the mitochondria through nutrient oxidation or the TCA cycle, where they can then act as cofactors to mitochondrial enzymes or be exported into the cytosol. The TCA cycle produces metabolites that contribute to epigenetic modification (Figure 2). For example, mutations to the TCA cycle enzyme isocitrate dehydrogenase (IDH) and production of 2-hydroxyglutarate (2HG) decrease α-ketoglutarate levels. The loss of α-ketoglutarate contributes to the decreased activity of lysine-specific demethylase 4C (KDM4C), causing an increase in H3K9 methylation that inhibits adipogenesis (Lu et al., 2012). In the following sections, we will discuss research that has illuminated how important metabolites contribute to epigenetic regulation in adipogenesis.

### Acetyl-CoA

Acetyl-CoA is a component of central carbon metabolism that provides carbon for lipogenesis. Beyond this role, acetyl-CoA is a donor group for histone acetylation *via* the activities of HATs (Shi and Tu, 2015). Within the mitochondria, acetyl-CoA is generated through β-oxidation of fatty acids, from pyruvate, and *via* branched-chain amino acid catabolism, and is reported to exit mitochondria through several mechanisms, including *via* the carnitine/acylcarnitine translocase and *via* SLC25A1 as citrate (Madiraju et al., 2009; Zaidi et al., 2012). Within the cytosol, the activity of ATP-citrate lyase (ACLY) converts citrate to acetyl-CoA and oxaloacetate. Cytosolic acetyl-CoA can take part in lipogenesis, steroidogenesis, or become a building block for glutamine, proline, and arginine (Pietrocola et al., 2015). Acetyl-CoA can also be regenerated from citrate in the nucleus by ACLY, driving a global increase in histone acetylation in 3 T3-L1 cells (Wellen et al., 2009). When acetyl-CoA is depleted following siRNA-mediated silencing of ACLY or following glucose restriction, histone acetylation levels are decreased, and adipocyte differentiation is inhibited (Wellen et al., 2009). However, some compensation has been observed as the loss of ACLY stimulates the expression of acyl-CoA synthetase short-chain family member 2 (ACSS2), which can generate acetyl-CoA from acetate (Zhao et al., 2016). High-fat diet leads to a decrease in whole-tissue acetyl-CoA levels in murine WAT and a subsequent decrease in H3K23ac, showing a correlation between diet, acetyl-CoA levels, and histone acetylation (Carrer et al., 2017). Elucidating the influence of diet on metabolite levels and histone alterations could have important implications for understanding obesity and associated diseases.

### NAD+

Metabolic pathways including the TCA cycle, ETC, fatty acid oxidation, and glycolysis influence the redox state of the cell through NAD+ metabolism. NAD+ is a small molecule that can be synthesized from precursors including nicotinamide (NAM), nicotinic acid (NA), and nicotinamide riboside (NR); through the *de novo* synthesis pathway from tryptophan; and through NAD+ salvage (Katsyuba et al., 2020). NAD+ acts as both a cofactor in redox reactions and a substrate for enzymes like poly [ADP-ribose] polymerases (PARPs) and sirtuins, which act as deacylases in the mitochondria, nucleus, and cytoplasm (Katsyuba et al., 2020). NAD+ is thought to exist in distinct mitochondrial, cytosolic, and nuclear compartments due to the presence of distinct nicotinamide mononucleotide adenylyltransferase (NMNAT) isoforms in these cellular locations (Berger et al., 2005; Nikiforov et al., 2011). NAD+ usage in these compartments causes fluctuations in nicotinamide mononucleotide (NMN) and NAD+ availability that have differing effects on epigenetic events that influence adipogenesis. Within the mitochondrial compartment, increased NAD+ production *via* increased flux through the TCA cycle is required to induce adipogenesis in 3T3-L1 cells (Okabe et al., 2020). Induction of adipogenesis increases the expression of cytosolic NMNAT2 resulting in elevated cytosolic NAD+ (Ryu et al., 2018). This activity of NMNAT2 decreases the availability of NMN for nuclear NMNAT1 and leads to a decline in nuclear NAD+. As nuclear NAD+ drops, PARP-1 activity decreases, leading to a subsequent reduction in its ADP-ribosylation of CEBPα. Loss of this modification increases the CEBPα proadipogenic transcriptional program (Ryu et al., 2018). Furthermore, NMNAT1 activity and synthesis of NAD+ direct PARP-1 ability to PARylate aspartate and glutamate residues on histones (Huang et al., 2020). These findings illustrate the temporal and compartment-specific influence of NAD+ metabolism on the cellular epigenetic state that modulates adipogenesis.

Compartment-specific alterations in NAD+ levels may also impact sirtuin activity. Increased mitochondrial NAD+ may promote the activity of SIRT4, an inducer of adipogenesis, while decreased nuclear NAD+ may decrease the activity of SIRT1, a negative regulator of adipogenesis (Mayoral et al., 2015; Zaganjor et al., 2021). NAD+ compartmentalization and availability may fine-tune adipogenesis *via* multiple independent mechanisms. SLC25A51 has been recently identified as a mitochondrial NAD+ transporter (Girardi et al., 2020; Kory et al., 2020; Luongo et al., 2020), which may present an opportunity to modulate NAD+ compartmentalization and cell differentiation.

### SAM, FAD, and α-Ketoglutarate

Other metabolites have been shown or suggested to influence epigenetic regulation, but their roles are not as well-defined. One such metabolite is S-adenosylmethionine (SAM). SAM acts as a major methyl donor and is utilized by histone lysine methyltransferases (HKMTs) as a cofactor, where the donation of a methyl group yields S-adenosylhomocysteine (SAH) (Wiese and Bannister, 2020). This metabolite is derived by S-adenosyl methionine transferase (MAT) by the catabolism of methionine and ATP or one-carbon metabolism (Wiese and Bannister, 2020). The addition of SAM to culture media induces differentiation in 3T3-L1 cells, although the precise mechanism has not been elucidated (Liu et al., 2013).

Another metabolite that participates in epigenetic regulation is flavin adenine dinucleotide (FAD). FAD is a cofactor utilized in redox reactions by demethylases, such as LSD1 and, once reduced, can be re-oxidized by molecular oxygen (Berger and Sassone-Corsi, 2016). Through coupling with FAD synthesis, LSD1 epigenetically regulates the expression of energy expenditure genes through the removal of mono- and di-methylation of H3K4, and knockdown of LSD1 results in activation of mitochondrial respiration and lipolysis in mature adipocytes (Hino et al., 2012). FAD levels increase both in differentiating 3T3-L1 preadipocytes as well as in mature 3T3-L1 cells following palmitate exposure to stimulate lipid storage and suppress PGC-1α (Hino et al., 2012). Alternatively, the knockdown of FAD-dependent LSD1 in differentiating 3T3-L1 cells leads to a significant decrease in lipid accumulation and expression of adipogenic regulator *Cebpa* (Musri et al., 2010). These findings suggest that FAD-dependent enzymes directly impact epigenetic regulation in adipocytes, indicating that FAD itself is a significant metabolite modulating adipogenesis.

Similarly, α-ketoglutarate acts as a cofactor for histone demethylases, as well as for DNA demethylation by TETs (Carey et al., 2015). Okabe et al. report that an increase in α-ketoglutarate is likely responsible for demethylation of H3K9me3 at the *Ppar*γ promoter, supporting adipogenesis (Okabe et al., 2020). These studies support the model that metabolites relay nutrient state information to alter gene expression and cell fate.

## Therapeutic Intervention

Adipogenesis is important in systemic metabolic health and increasing adipogenesis may profoundly improve outcomes for patients with obesity-related diseases, such as diabetes. Mutations in genes that regulate lipid droplet formation lead to lipodystrophy, a condition characterized by severe IR and dyslipidemia (Magre et al., 2001; Agarwal et al., 2002; Ghaben and Scherer, 2019). Inhibition of adipogenesis through the depletion of PPARγ in the progenitor population results in pathological WAT expansion in mice (Shao et al., 2018). Collectively, these studies suggest the protective effects of adipogenesis and warrant the search for therapeutic interventions that promote the process. Thiazolidinediones (TZDs), synthetic PPARγ activators, although promising in their ability to promote adipogenesis, adipose tissue beiging, insulin sensitivity and reduced inflammation, also carry reported cardiac and osteoporosis risks (Ahmadian et al., 2013). Evaluating metabolic targets in their ability to induce adipogenesis in the context of diet-induced obesity may have therapeutic benefits. Notably, BCAAs accumulate in diabetic patients and BCAA catabolism in adipose tissue is dysfunctional in diabetic mouse models (Felig et al., 1969; Newgard et al., 2009; Neinast et al., 2019b). Identifying small molecules that potentiate this pathway may improve adipogenesis and overall health. Similarly, high-fat diet downregulates ACLY in WAT, alters histone acetylation, and disrupts lipid storage (Carrer et al., 2017). Future studies are necessary to evaluate the impact of maintaining ACLY levels on adipogenesis and metabolic health after a dietary challenge. Recent technological advances in mass spectrometry, compartmentalized metabolomics and general growth of the field will allow for discoveries on new regulators of adipose development (Chen et al., 2017; Collins et al., 2021). Elucidating the metabolic drivers of adipogenesis may lead to novel approaches to mitigate obesity-mediated disorders.

## Author Contributions

All authors listed have made a substantial, direct, and intellectual contribution to the work and approved it for publication.

## Funding

EZ was supported by the Vanderbilt Digestive Diseases Research Center Grant (P30 058404).

## Conflict of Interest

The authors declare that the research was conducted in the absence of any commercial or financial relationships that could be construed as a potential conflict of interest.

## Publisher’s Note

All claims expressed in this article are solely those of the authors and do not necessarily represent those of their affiliated organizations, or those of the publisher, the editors and the reviewers. Any product that may be evaluated in this article, or claim that may be made by its manufacturer, is not guaranteed or endorsed by the publisher.
